# A Prospective Cross-Sectional Study of Acute Coronary Syndrome Patients’ Quality of Life and Drug Prescription Patterns at Riyadh Region Hospitals, Saudi Arabia

**DOI:** 10.3390/healthcare11131973

**Published:** 2023-07-07

**Authors:** Mohamed F. Balaha, Ahmed A. Alamer, Ahmed M. Kabel, Saad A. Aldosari, Sarah Fatani

**Affiliations:** 1 Department of Clinical Pharmacy, College of Pharmacy, Prince Sattam Bin Abdulaziz University, Al-Kharj 11942, Saudi Arabia; 2 Pharmacology Department, Faculty of Medicine, Tanta University, Tanta 31527, Egypt

**Keywords:** acute coronary syndrome, health-related quality of life, drug prescription pattern, Saudi Arabia, MRCB scale, GRACE score, TIMI score

## Abstract

Acute coronary syndrome (ACS) is a leading cause of cardiovascular-related morbidity and mortality worldwide. The present study investigated the health-related quality of life (HRQOL) and drug prescribing patterns in ACS patients at Riyadh hospitals in Saudi Arabia. This study was a 12-month prospective cross-sectional study that included 356 patients with ACS. The current study showed that younger male (67.42%) and urban (75.84%) patients suffered more from ACS. Moreover, most patients with NSTEMI (51.69%) experienced Grade 1 dyspnea (33.43%) and NYHA Stage 2 (29.80%); however, STEMI patients were at greater mortality risk. The HRQOL questionnaire showed that ACS patients were significantly impaired in all QOL domains (emotional [23.0%, *p* = 0.001], physical [24.4%, *p* = 0.003], and social [27.2%, *p* = 0.002]). Furthermore, the most commonly prescribed medications were statins (93%), antiplatelets (84%), anticoagulants (79%), coronary vasodilators (65%), and beta-blockers (63%). Additionally, 64% of patients received PCIs or CABGs, with the majority of cases receiving PCIs (49%), whereas 9% received dual anticoagulant therapy. Thus, there is an urgent need to educate healthcare teams about the relevance of QOL in ACS control and prevention and the new ACS management recommendations. ACS is also growing among younger people, requiring greater attention and prevention.

## 1. Introduction

According to the World Health Organization, cardiovascular diseases (CVDs) are the leading cause of mortality globally, claiming an estimated 17.9 million lives per year [1]. In addition, in the past four decades, ischemic heart disease (IHD) has been responsible for one-third of all deaths [2]. In addition, according to a report issued by the World Health Organization in 2012, around 7.4 million fatalities were caused by IHD, accounting for 42% of cardiovascular-related deaths and 13% of global mortality [3]. Moreover, the American Heart Association’s (AHA) 2023 Heart-Disease and Stroke Statistics Update documented that about 15.5 million people in the United States were diagnosed with IHD, and the related cost was 207 billion dollars [4]. Furthermore, between 2013 and 2030, this cost is anticipated to climb by 43% [5]. In Saudi Arabia, IHD has a substantial economic burden (10,710 dollars per patient) and has been the top cause of death from 2000 to 2012, with a mortality rate of 21.7% in 2012 [6]. In addition, Saudi Arabia is undergoing a significant revolution as a young country that has adopted a Western lifestyle, with a rise in the incidence of CVD risk factors [7].

IHD is subdivided into coronary artery disease (CAD), silent myocardial ischemia, and acute coronary syndrome (ACS) [6,8]. While CAD can be asymptomatic, ACS is characterized by signs and symptoms of abrupt myocardial ischemia in the presence of stable CAD. CAD is caused by atherosclerosis in the coronary arteries, in which atherosclerotic plaque builds up inside the coronary arteries, restricting blood flow and, thereby, oxygen delivery to the heart [5,9]. In addition, ACS is a major cause of the global burden of CVS, contributing to 18 million deaths in 2016, and an estimated 23 million people will die by 2030. The recurrence of ACS is also very common, with 40% of survivors being readmitted to the hospital within 30 days after discharge and 20% experiencing a second cardiac event within the first year [9,10]. It has a significant economic and morbidity impact on the patient, his family, and society [7]

The term “ACS” refers to patients who are suspected of experiencing acute myocardial ischemia or infarction as a result of a dramatic reduction in coronary blood flow. There are three forms of ACS: ST-elevation myocardial infarction (STEMI), non-ST-elevation myocardial infarction (NSTEMI), and unstable angina (UA) [11]. Every year, around 8 million people in the United States seek treatment in emergency rooms for chest pain. Over 1.5 million people are admitted to hospitals with an ACS each year (330,000 with STEMI and 1.24 million with UA and NSTEMI) [12]. Furthermore, according to a prior single-center study conducted in Saudi Arabia, the leading reason for coronary care unit (CCU) admission was ACS [13]. Myocardial infarction (MI) is the permanent damage of heart muscle caused by a prolonged lack of oxygen flow. However, unstable angina is employed when there is myocardial ischemia but no obvious myocardial necrosis [14].

Although atherosclerotic plaque rupture and infarct-related arterial thrombosis are the most common causes of ACS, stable CAD can advance to ACS without plaque rupture and thrombosis if physiologic stress increases the burden on the heart [15]. Chest discomfort that presses, squeezes, or burns and spreads to the neck, shoulder, arm, jaw, back, or upper abdomen is the most prevalent ACS symptom. Sweating, nausea, vomiting, dyspnea, palpitations, fainting, anxiety, restlessness, a sudden blood pressure drop, and death are further symptoms [12]. ACS patients, particularly MI patients, have an 80% chance of survival after a coronary event, but the youngest survivors are high-risk and require long-term preventative measures to enhance their quality of life [16].

ECG and symptoms determine diagnosis and therapy. ST-elevation in the leads indicates STEMI, while peaked upright or inverted T-waves indicate acute myocardial damage and early transmural Q-wave MI. Persistent ST depression may indicate non-Q-wave MI [17]. Cardiovascular markers predict ACS best (troponin and creatine kinase MB isoform). Acute ischemia is indicated early by troponin and CK-MB. Cardiac troponin levels rise 3–12 h after symptoms start, peak at 24–48 h, and return to baseline in 5–14 days. CK-MB levels rise 3–12 h after the pain starts, peak in 24 h, then return to baseline in 48–72 h [18].

Prehospital care may include oxygen, aspirin, and nitrates, as well as hospital transport. Anticoagulants, antiplatelets, and antianginals are among the therapeutic drugs utilized. In challenging cases, angiography may be used to examine the structure of the coronary artery. Reperfusion therapy consists of fibrinolysis, percutaneous coronary intervention (PCI), and coronary artery bypass grafting (CABG). Typically, STEMI patients need immediate reperfusion treatment, such as PCI, CABG, or fibrinolytic therapy. Dual anticoagulant medicines may be required for early invasive therapy in patients with NSTEMI. Conservative therapy with a single anticoagulant may be utilized for unstable angina [19,20].

Several dimensions of the patient’s quality of life (QOL) may be compromised by coronary heart disease, including physical, psychological, emotional, and social dimensions. Physical activity is the most impacted, followed by emotional and social factors. The measurement of health-related quality of life (HRQOL) is an essential patient-centered health outcome that may be used to evaluate the impact of disease burden and the efficacy of treatment strategies [21]. The severity of a patient’s angina/MI/CAD impacts their functional level and risk of cardiovascular consequences and is thus critical to the successful treatment of the illness. HRQOL measurements evaluate a patient’s experience with health problems in areas such as physical, emotional, and social functioning, performance role, pain, and tiredness. A poor HRQOL score can have an impact on the process of recovery, decrease treatment adherence, diminish the capacity to conduct activities of daily living, raise hospital readmission rates, and place the patient at risk for complications and mortality [5,11].

The MacNew’s Heart Disease HRQOL Questionnaire is a self-administered questionnaire that assesses current angina symptoms as well as the impact of CAD therapy on daily activities and physical, emotional, and social functioning [22]. The Global Registry of Acute Coronary Events (GRACE) Score estimates the risk of death or death/MI in patients following the initial ACS. The STEMI Thrombolysis in Myocardial Infarction (TIMI) Risk Score is a basic risk assessment score that may be utilized at the bedside. In patients with NSTEMI and unstable angina, the TIMI score predicts ischemic events and mortality [11,23]. However, the Medical Research Council’s Breathlessness (MRCB) scale and the New York Heart Association (NYHA) scale were used to assess the patient’s physical affection [11].

In recent years, medication prescribed based on practice recommendations has improved to promote proper therapeutic intervention for ACS secondary prevention. Statins, dual antiplatelet agents, angiotensin-converting enzyme inhibitors (ACEI) or angiotensin receptor blockers (ARB), beta-blockers, and nitroglycerin, are all recommended medications for individuals with ACS. In addition, risk-based treatment is essential for ACS. Moderate- to high-risk patients are admitted to a coronary critical care unit based on symptoms and risk. High-risk patients should have coronary angiography and PCI or CABG within 24–48 h of significant coronary artery stenosis. During hospitalization, moderate-risk individuals with biochemical signs of infarction usually undergo angiography and revascularization [5,24].

Saudi Arabia’s 2030 vision is to enhance healthcare systems by collecting appropriate data in order to meet and manage challenges, implement preventative measures, and increase access to healthcare systems. Moreover, Saudi Arabia is divided into regions, each with its own patient demographics, clinical features, management, and quality of healthcare services [7,25]. As a result, the current study’s objectives seek to assess the quality of life and drug prescription patterns of ACS patients at Riyadh Region Hospitals in Saudi Arabia. Investigating these factors may aid in the implementation of more effective approaches to the prevention and management of ACS, as well as potentially improving healthcare systems and thereby improving HRQOL in ACS patients.

## 2. Materials and Methods

### 2.1. Study Design and Participants

Between 1 January 2022 and 31 December 2022, a 12-month prospective cross-sectional study was conducted in the cardiology departments of Riyadh region hospitals in Saudi Arabia. This study was carried out following the Helsinki Declaration and was authorized by the institutional review board of the Standing Committee of Bioethics Research at Prince Sattam bin Abdulaziz University (SCBR-081-2021). This study comprised male and female patients who matched the following inclusion criteria: they were above the age of 18, had evidence of UA, NSTEMI, or STEMI, were receiving medication and/or revascularization, and were willing to participate. Patients with congenital cardiac defects (such as coarctation of the aorta and transposition of the great arteries), unstable hemodynamic circumstances (such as unconscious patients, uncontrolled hypertension [systolic blood pressure > 180 mmHg and/or diastolic blood pressure > 100 mmHg, resting heart rate > 40 beat per minutes, significant arrhythmias and significant renal and hepatic failure), or ACS caused by coronary artery dissection, stress-induced cardiomyopathies, endocarditis, and a non-cardiovascular cause, such as anemia, coronary embolus, and surgery (such as patients with operated coronary arteries in the infancy or with a previous history of cardiopulmonary operations), were excluded. This research included 356 people who had been identified with ACS.

### 2.2. Study Tools

The HRQOL MacNew Heart Disease questionnaire, the Medical Research Council Breathlessness, the NYHA Functional Scales, GRACE, and TIMI Scores were used in this investigation.

The QOL of ACS patients was assessed using the HRQOL MacNew Heart Disease questionnaire, the MRCB, and the NYHA Functional scales. The HRQOL MacNew Heart Disease questionnaire has 27 items spread over three primary domains (physical, emotional, and social), each with a Likert scale ranging from 1 to 7. The tool’s global score ranges from 7 to 189. Following that, the mean of the global score was calculated, with a score of one indicating poor QOL and a score of seven indicating excellent QOL [26]. The MRCB scale classifies shortness of breath in patients as Grades 0, 1, 2, 3, and 4. Whereas Grade 0 indicates no shortness of breath except during intense exercise, Grade 1 indicates that shortness of breath occurs when hurrying on level ground or walking up a slight uphill, Grade 2 indicates walking slower than most people on level ground, stopping after a mile or so, or stopping after 15 min of walking at your own pace, Grade 3 indicates stopping for breath after walking about 100 yards or after a few minutes on level ground, and Grade 4 indicates severe shortness of breath at minor efforts such as dressing or undressing [27]. However, the NYHA scale classifies patients based on their physical ability into four Stages: 1, 2, 3, and 4, with Stage 1 indicating no limitation to ordinary physical activity, Stage 2 indicating mild breathlessness and fatigue, and slight limitation during ordinary activity, Stage 3 indicating a marked limitation of physical activity due to breathlessness and fatigue even during less-than-ordinary activity, and Stage 4 indicating severe symptoms even at rest [28].

The GRACE and TIMI risk scores were used to predict mortality in STEMI and NSTEMI patients. The GRACE score was calculated by adding the points for each of the following eight prognostic variables: age, history of heart failure, history of acute myocardial infarction, heart rate and SBP at admission, ST-segment depression, serum creatinine, and elevated myocardial necrosis markers or enzyme. Age, aspirin usage in the previous week, number of angina episodes in the previous 24 h, high blood myocardial necrosis markers or enzymes, ST-segment deviation, history of coronary artery disease, and the presence of three risk factors for heart disease all contribute to the TIMI risk score for patients with NSTEMI. Low-risk individuals have scores ranging from 0 to 2, intermediate-risk patients have scores ranging from 3 to 4, and high-risk patients have scores ranging from 5 to 7 [11,23]. However, the TIMI risk score for STEMI patients was determined by adding points for the following factors: patient age, presence of diabetes, hypertension, or angina, systolic blood pressure < 100 mmHg, heart rate > 100 beats per minute, Killip Class II-IV, weight < 67 kg, anterior ST elevation or LBBB, and time to treatment > 4 h. Where, Score 0: 30 Day Mortality is 0.8%, Score 1: 30 Day Mortality is 1.6%, Score 2: 30 Day Mortality is 2.2%, Score 3: 30 Day Mortality is 4.4%, Score 4: 30 Day Mortality is 7.3%, Score 5: 30 Day Mortality is 12.4%, Score 6: 30 Day Mortality is 16.1%, Score 7: 30 Day Mortality is 23.4%, Score 8: 30 Day Mortality is 26.8%, and Score > 8: 30 Day Mortality is 35.9% [29].

### 2.3. Data Collection

During the first meetings and follow-up interviews at hospital cardiology clinics, data were collected. After gaining permission from the participants, the author of the study assessed exclusion criteria, obtained informed consent from interested and eligible patients, and set up a 6-month meeting. The interview was structured to ensure consistency in collecting data. In addition, every effort was made to protect the privacy and confidentiality of participant data. The research data collection format and the interview protocol were checked and confirmed by hospital preceptors. Patients provided signed informed consent for the collection of data. The form is used to capture sociodemographic data (age, gender, ethnicity, social background, past history, and family history of CVS problems), symptoms, diagnosis, medicines prescribed, and surgical procedures. Data from the MacNew Heart Disease questionnaire, the MRCB, the NYHA functional scales, GRACE, and TIMI scores were compiled using an Excel data collection sheet.

### 2.4. Statistical Analysis

IBM-SPSS Statistics Software for Windows (IBM-SPSS, version 25, Armonk, NY, USA) was used to perform a descriptive analysis of the data to determine the mean and standard deviation. The Chi-square test was performed to determine the significance between various data acquired for each risk factor variable and each HRQOL domain. The *p*-value is utilized to measure statistical significance within statistical hypothesis significance for the evaluation of HRQOL in ACS patients compared to the baseline visit using statistical hypothesis significance. The *p*-value was set to 0.05, and the confidence interval was set at 95%.

## 3. Results

### 3.1. The Sociodemographic, Symptoms, and Diagnostic Profiles of ACS Patients

Based on our inclusion criteria, 356 patients were included in this study. Table 1 depicts the sociodemographic characteristics of ACS patients. Individuals aged 48 and higher were substantially more likely to get ACS. Males were more likely to develop ACS. Furthermore, urban inhabitants developed ACS at a higher rate than rural residents. The majority of patients had ACS risk factors. Smoking was present in 27.03 percent of patients, and 32.3 percent had a family history of cardiovascular disease. 47.47 percent were hypertensive, 37.64 percent were diabetic, 12.08 percent had neuropsychological issues, 7.02 percent had renal disorders, and 5.9 percent were respiratory. Chest pains (82.87 percent), shortness of breath (42.98 percent), and sweating (29.21 percent) were the most common symptoms. Other issues include edema and cough (17.7 percent), as well as palpitations and vomiting (15.73 percent). NSTEMI (51.69 percent) was substantially more prevalent than STEMI (29.49 percent) and unstable angina (18.82 percent). Most patients (49.16 percent) belonged to the NYHA Stage 2 functional class, as compared to Stage 1 (29.49 percent) and Stage 3 (23.88 percent). The vast majority of patients (33.43 percent) have Grade 1 and Grade 2 SOB (28.93 percent).

### 3.2. The MacNew Heart Disease Health-Related Quality of Life Questionnaire

All research participants received the MacNew HRQOL questionnaire. Table 2 summarizes their emotional state, physical functioning, and social dependence. A total of 11 percent of the patients had strong emotional functioning, 29.5 percent had good emotional functioning, 25.3 percent had fair emotional functioning, and 23.0 percent had poor emotional functioning. Furthermore, 47.5 percent of patients had mild physical symptoms, 28.1 percent reported moderate physical symptoms, and 24.4 percent reported severe physical symptoms. However, 27.2 percent of the patients need social support.

### 3.3. Mortality Risk in Hospital and at 6 Months Calculated Using GRACE Score

Table 3 shows the GRACE score-based mortality risk of hospitalized patients. It shows that 11.2 percent of patients were at high risk, 21.3 percent were at intermediate risk, and 67.4 percent were at low risk. We also looked at the six-month mortality risk of patients and discovered that 34.8 percent were high-risk, 50.6 percent were intermediate-risk, and 14.6 percent were low-risk. The majority of individuals in our dataset had an intermediate mortality risk according to the GRACE risk score at 6 months (50.6 percent).

### 3.4. Mortality Risk in NSTEMI and STEMI Patients Calculated Using TIMI Score

The calculated percentage of mortality risk using the TIMI score is shown in Table 4. According to the TIMI score, 65 percent of 184 NSTEMI patients and 51.43 percent of 105 STEMI patients had a low risk of death; however, 14.29 percent of 105 STEMI patients had a high risk of mortality.

### 3.5. Diagnosis versus NYHA Functional Class

Table 5 depicts the diagnosis versus NYHA functional class. A total of 19 percent of NSTEMI patients were classified as NYHA Stage 1, 57.6 percent as NYHA Stage 2, and 23.4 percent as NYHA Stage 3. In addition, 20.0 percent of STEMI patients were classified as having NYHA Stage 1, 47.6 percent as having Stage 2, and 32.4 percent as having Stage 3. In addition, 59.7 percent of angina patients had NYHA Stage 1, 28.4 percent had NYHA Stage 2, and 11.9 percent had NYHA Stage 3. The majority of patients with ACS (49.16 percent) were NYHA Stage 2. The present study revealed that NSTEMI and STEMI patients were considerably less able to engage in physical activity compared to angina patients.

### 3.6. Diagnosis versus MRCB Scale

As shown in Table 6, patients were classified based on the MRCB scale and their diagnosis. Totals of 1.90 percent of STEMI patients, 11.41 percent of NSTEMI patients, and 38.81 percent of all patients had Grade 0 SOB (13.76 percent of total patients). Grade 1 SOB was seen in 24.76 percent of STEMI patients, 38.59 percent of NSTEMI patients, and 32.84 percent of angina patients (33.43 percent of total patients). Grade 2 SOB was found in 37.14 percent of STEMI patients, 32.61 percent of NSTEMI patients, and 5.97 percent of angina patients (28.93 percent of total patients). Grade 3 SOB was found in 15.24 percent of STEMI patients, 11.41 percent of NSTEMI patients, and 22.39 percent of angina patients (14.61 percent of total patients). Grade 4 SOB was detected in 20.95 percent of STEMI patients and 5.98 percent of NSTEMI patients (9.27 percent of total patients). The vast majority of individuals had Grade 1 or 2 SOB.

### 3.7. Classes of Commonly Prescribed Medicines and Surgical Interventions for ACS Patients

The classes of commonly prescribed medicines and surgical interventions for ACS patients are outlined in Table 7. Commonly prescribed drug groups were statins (93 percent), antiplatelets (84 percent), anticoagulants (79 percent), coronary vasodilators (65 percent), beta-blockers (63 percent), diuretics (38 percent), ARBs (31percent), ACE Inhibitors (22 percent), calcium channel blockers (CCBs, 20 percent), and inotropes (in acute heart failure patients, 7 percent). In addition, 64 percent of patients have surgical interventions, and the majority of patients (49 percent) undergo reperfusion therapy with PCI as opposed to CABG (15 percent).

Simvastatin was the most often prescribed statin, comprising 39 percent of all given statins. In addition, rosuvastatin contributes 33 percent, atorvastatin contributes 18 percent, and other statins contribute 10 percent. 

Aspirin was the most often prescribed antiplatelet drug (42 percent). However, 32 percent administered clopidogrel, 5 percent administered ticagrelor, 3 percent administered tirofiban, and 2 percent administered additional antiplatelet medicines. In addition, twelve percent of patients were provided dual antiplatelet medications, and four percent were prescribed triple antiplatelet medications.

Heparin was the most common anticoagulant administered (57 percent). In addition, 25 percent of all prescriptions are filled with enoxaparin, 7 percent with rivaroxaban, and 2 percent with other anticoagulants. A total of nine percent of patients nevertheless received dual anticoagulant medication.

Nitrates were the most often recommended coronary vasodilators (78 percent). In addition, amlodipine accounted for eighteen percent of prescriptions, and trimetazidine accounted for three percent of prescriptions. One percent of prescriptions were for additional coronary vasodilators.

Metoprolol was the most often recommended beta-blocker (50 percent). In addition, 42 percent of prescriptions had bisoprolol, 6 percent contained carvedilol, and 2 percent contained additional beta-blockers. 

Furosemide was the most often prescribed diuretic (52 percent). In addition, 24 percent of prescriptions contain torsemide, 13 percent involve hydrochlorothiazide, 11 percent involve spironolactone, and 1 percent involve other diuretics. 

Valsartan was the ARB most frequently prescribed (40 percent). Moreover, 26 percent of prescriptions include telmisartan, 19 percent involve losartan, 13 percent involve irbesartan, and 2 percent involve additional ARBs.

Forty-five percent of ACE inhibitor prescriptions were for captopril. In addition, 42 percent of prescriptions contain lisinopril, 10 percent involve perindopril, 2 percent involve quinapril, and 1 percent involve other ACE inhibitors.

Amlodipine was most frequently administered among CCBs (76 percent). Additionally, 18 percent of prescriptions involve verapamil, 4 percent involve nifedipine, and 2 percent involve other CCBs.

In cases of acute heart failure with ACS, dobutamine (63 percent) was employed more frequently than noradrenaline (33 percent) and dopamine (4 percent), according to the findings of the current study.

For patients with hypertension (47.47 percent), the beta-blocker (43 percent) was the most often recommended medicine alone, followed by ACE inhibitors (28 percent), ARBs (16 percent), CCBs (9 percent), and diuretics (4 percent). The majority of patients (85 percent), however, were prescribed a combination treatment, with the most common combination being beta-blockers + ACE inhibitors (33 percent), followed by beta-blockers + ARBs (29 percent), beta-blockers + CCBs (22 percent), beta-blockers + diuretics (10 percent), and other combinations (6 percent).

## 4. Discussion

Acute coronary syndrome (ACS) is a leading cause of cardiovascular-related morbidity and mortality worldwide. The present study investigated the HRQOL and drug prescribing patterns in ACS patients at Riyadh hospitals in Saudi Arabia. The study was a 12-month prospective cross-sectional study between 1 January 2022 and 31 December 2022 that included 356 patients with ACS. The current study showed that younger males and urban patients suffered more from ACS. Moreover, most patients had NSTEMI, experienced Grade 1 dyspnea, and were NYHA Stage 2; however, STEMI patients were at greater mortality risk. The HRQOL questionnaire showed that ACS patients were significantly impaired in all QOL domains (emotional, physical, and social). Furthermore, the most commonly prescribed medications were statins, antiplatelets, anticoagulants, coronary vasodilators, and beta-blockers. Additionally, 64% of patients received PCI or CABG, with the majority of cases receiving PCI (49%), whereas 9% received dual anticoagulant therapy.

Individuals aged 48 and higher were substantially more likely to get ACS. A previous study conducted in Saudi Arabia’s northern area reported that the majority of ACS cases were discovered among persons aged 46 to 55, which is similar to our findings [25]. Contrary to our result Al-Saif et al., 2012, documented that the mean age of ACS in Saudi Arabia is 58 years [30]. This contradiction could be due to a variety of factors, including a lack of evaluation by healthcare workers and a lack of primary care physicians’ understanding of treatment, advanced therapies, new technologies to aid in the management of cardiovascular risk factors, and an increase in smoking and obesity in the younger age population. Furthermore, as a young nation, Saudi Arabia is undergoing a tremendous shift with the adoption of a western lifestyle, which has resulted in a rise in the incidence of cardiovascular disease risk factors [7,31]. All the aforementioned variables may have a role in poor risk factor management and the beginning of ACS at a younger age.

Aging and inflammation cause the creation of atherosclerotic plaques, which include the establishment of a necrotic core, fibrous cap, matrix thickening, and plaque instability. Furthermore, increasing age was related to lower baseline renal function, which is a predictor of poor prognosis in patients with ACS. This process results in STEMI, NSTEMI, and UA, with this patient having an imbalance between oxygen demand and supply [12]. As a result, older people have more calcification, as well as more multivessel and major diseases. Similarly, 49.7 percent of our patients are over 50, and 69.2 percent are over 40. In other words, rising age reduces HRQOL’s physical health. Furthermore, Andhi et al. (2022) suggested that older age might predict poorer HRQOL in ACS patients [11]. Interestingly, another study found no correlation between age and quality of life. This discrepancy relies on whether or not patients are participating in a cardiac rehabilitation program [32]. Consequently, the health service provider should encourage the enrollment of ACS patients in rehabilitation settings. In addition, further research must be conducted on the HRQOL components of ACS patients.

The current study’s population was made up of 67.42 percent men and 32.58 percent women. According to AHA, the American Heart Association’s (AHA) 2023 Heart-Disease and Stroke Statistics Update, the incidence of MI was greater in males than in women [4]. Furthermore, another study in Saudi Arabia’s southern region discovered that the frequency of ACS was greater in males than in females. This shows that because of the high incidence of dyslipidemia, smoking, hypertension, and diabetes among men; thus they are at a greater risk of developing ACS [25]. On the contrary, Thiruvisaakachelvy et al., 2019 revealed that there was no significant correlation between gender and ACS patients’ life quality due to the improved health care and rehabilitation services they received [32]. In our study, the proportion of urban people diagnosed with ACS (75.84 percent) appears to be higher than the proportion of rural people (24.16 percent). According to AHA publications, the global burden of cardiovascular illnesses in urban populations has increased relative to the incidence of heart disease due to increased intake of animal protein and fats, as well as decreasing physical activity. Recent research in Saudi Arabia demonstrated that urban individuals are more impacted by ACS than rural people. This might be due to significant consequences for healthcare services and resource use, as well as the accessibility of healthcare facilities in urban areas vs rural areas [25].

In our 356-patient research study, 26.97% were smokers, and 32.30% had a family history of coronary artery disease (CAD). Saudi Arabia has a lower proportion of current smokers than the Gulf RACE registries, with 66 percent of persons under 40 being current smokers. Other polls indicate that younger generations are adding to the pool of smokers [7,30]. Moreover, Balgaith et al. (2017) also discovered a 60% smoking rate among young Saudis with ACS [33]. This was comparable to the findings of the GRACE trial, which demonstrated an association between cigarette smoking and STEMI in young patients with ACS [34]. Cigarette smoking is a known cause of endothelial dysfunction; it can also produce vasoconstriction, promote atherosclerosis, and generate a thrombotic state [35]. Comorbid diseases were seen in 79% of the 356 individuals. A total of 47.47 percent were hypertensive, 37.64 percent were diabetic, 12.08 percent were neuropsychological disorders, 7.02 percent were renal diseases, and 5.9 percent were respiratory problems. The AHA’s 2023 Heart Disease and Stroke Statistics Update indicated equivalent results [4]. Al-Saif et al. (2012) documented in another study conducted in 2012 that the rate of DM was 58 percent in all studied patients, with 26 percent being younger than 40 years old [30]. The prevalence of diabetes in the Saudi Arabian population over the age of 25 was 23.7 percent in 1995, 30.8 percent in 2013, and 39.5 percent by 2022, which might be attributed to an increase in obesity prevalence, genetic and environmental factors. Similarly, hypertension afflicted 26 percent of the population in Saudi Arabia in 1995, rising to 25.5 percent in 2005 and 28 percent in 2013 [25,36,37]. The comorbidities of diabetes mellitus and hypertension with ACS worsen the morbidity and mortality of ACS patients, as well as their quality of life. To reduce morbidity and mortality associated with hypertensive and diabetic patients, the healthcare system must adhere to stringent control protocols [38].

Symptoms are the impetus for patients with symptoms suggestive of acute coronary syndrome (ACS) to seek emergency treatment for this potentially life-threatening disorder [39]. The most common patient complaints in our study were chest pain (82.87 percent), shortness of breath (42.98 percent), and sweating (29.21 percent). Other symptoms include edema, coughing (17.7 percent) and palpitations with vomiting (15.73 percent). Among the 356 patients, 184 (51.69 percent) were diagnosed with NSTEMI, 105 (29.49 percent) with STEMI, and 67 (18.82 percent) with angina. Andhi et al., 2022 and Saju et al., 2020 found that the majority of patients had chest pain as the primary symptom, followed by shortness of breath and profuse perspiration, which was consistent with our findings [5,11]. Thus, patients with ACS risk factors must be educated by healthcare providers to seek emergency treatment when experiencing these symptoms.

We used the GRACE score to assess the mortality risk rate of patients at 6 months and discovered that 34.8 percent were at high risk, 50.6 percent were at intermediate risk, and 14.6 percent were at low risk. At 6 months, the vast majority of our patients had an intermediate risk of mortality according to the GRACE risk score (50.6 percent). The TIMI score was also used to quantify the proportion of mortality risk. According to the TIMI score, 65 percent of 184 patients diagnosed with NSTEMI and 51.43 percent of 105 patients diagnosed with STEMI had a low risk of death; however, 14.29 percent of 105 patients identified with STEMI had a high risk of mortality. Andhi et al., 2022 and Wong & White, 2005 show comparable results to our study, with the proportion of high-risk patients based on the GRACE and TIMI scores being 36 percent and 57 percent, respectively [11,40]. Hence, the performance of dynamic risk assessment scales with serial evaluations is crucial for identifying patients who require more intense therapy.

Nineteen percent of NSTEMI patients were classified as NYHA Stage 1, 57.6 percent as NYHA Stage 2, and 23.4 percent as NYHA Stage 3. Furthermore, 20 percent of STEMI patients were classified as NYHA Stage 1, 47.6 percent as NYHA Stage 2, and 32.4 percent as NYHA Stage 3. Furthermore, 59.7 percent of angina patients had NYHA Stage 1, 28.4 percent had NYHA Stage 2, and 11.9 percent had NYHA Stage 3. The vast majority of ACS patients (49.16 percent) were in NYHA Stage 2. Andhi et al., 2022 reported results that were comparable to ours [11].

There has been a lot of focus in recent years on the negative impact of depression on outcomes in individuals with CHD [41]. QOL is also an indicator of the quality of health care and is a type of treatment program. Measuring QOL in chronic illnesses provides more information to the treatment team regarding the health of the patients [42]. Healthcare practitioners should focus not just on the physical aspect of patients but also on their whole quality of life in terms of physical, psychological, mental, and social well-being [15,25]. According to our results, the ability to engage in physical activity was significantly diminished in NSTEMI and STEMI patients. Similar to this study, Andhi et al., 2022 and Imam & Jitpanya, 2022 discovered that 45 percent of individuals did not reach the physical activity requirements after ACS diagnosis [11,16]. Moreover, lower HRQOL is frequently reported following cardiac incidents. Persistent symptoms, both physical (e.g., pain and tiredness) and psychological (e.g., sadness and anxiety), lower patients’ perceived degree of personal competence and capacity to accomplish daily tasks. ACS survivors are 2.7 times more likely than the general population to have fair/poor general health and 1.5 times more likely to experience limits in daily activities [15,22]. Accordingly, more extensive interventions are necessary to improve patients’ quality of life, so boosting physical functioning, reducing physical constraints, and restoring former skills, thereby reducing morbidity and mortality among ACS patients.

Twenty-seven percent of 356 patients received conservative therapy (a single anticoagulant), nine percent had aggressive therapy (dual anticoagulants), and sixty four percent underwent surgical procedures such as PCI or CABG (reperfusion therapy). According to a previously published study, PCI was the most suitable strategy for patients with STEMI since the majority of patients over the age of 60 performed the procedures, which improves QOL [43]. PCI was employed as both a diagnostic and therapeutic technique, emphasizing how important this intervention is in situations of myocardial infarction. It is crucial to mention that PCI is also utilized as a therapeutic option for patients with ACS since inserting a stent into the coronary arteries reduces the risk of death by around 30% [44]. Compared to the rates attained in the ACTION (81%) and GRACE (58%) registries, the overall rate of PCI was quite low [45]. Consequently, it is crucial to expand the number of healthcare institutions supplied with PCI facilities. Furthermore, since its inception in the 1960s, CABG has shown gradual improvements in reducing disparities, with the use of in situ internal mammary artery (IMA) grafting having the most dramatic favorable effect [41]. This is comparable to earlier studies conducted by Andhi et al., 2022; Saju et al., 2020; and Xavier et al., 2008, which showed growing trends of PCI in ACS, demonstrating that early perfusion is crucial to the management of STEMI patients [5,11,46].

Medication utilization research provides a more accurate assessment of reasonable drug use. Antiplatelets, anticoagulants, antidyslipidemics, antihypertensives, antianginals, beta-blockers, diuretics, ARBs, ACE, CCBs, and inotropes are some medications used to treat ACS. These medications were used to lower cardiovascular mortality and nonfatal myocardial infarction in patients with ACS [5,11]. Statins (93 percent), antiplatelets (84 percent), anticoagulants (79 percent), coronary vasodilators (65 percent), beta-blockers (63 percent), diuretics (38 percent), angiotensin-converting enzyme (ACE) inhibitors (22 percent), CCBs (20 percent), and inotropes (in acute heart failure patients, 7 percent) were the most commonly prescribed drug groups in our study. This is consistent with the literature since statins are the most widely prescribed drugs for dyslipidemia in the treatment of persons with ACS. Statin therapy reduces the incidence of CAD, recurrent MI, and stroke in patients who have been stabilized following ACS. This finding is consistent with the current guidelines for using medication in cardiovascular crises [5,47]. Furthermore, Al-Saif et al. (2012) found that the overall usage of statins following discharge was 93 percent and was unaffected by age. The usage of beta-blockers dropped with age, reaching an all-time high of 85 percent in patients over the age of 40, while the use of ACEI or ARB grew with age but did not surpass 78 percent in those over the age of 56 [30].

Aspirin was the most commonly prescribed antiplatelet drug (42 percent). Nonetheless, 32 percent were given clopidogrel, 5 percent were given ticagrelor, and 3 percent were given tirofiban. Similar findings were previously recorded by Andhi et al., 2022 and Al-Saif et al., 2012 [11,30]. According to a meta-analysis conducted by the Antithrombotic Trialists Collaboration, the use of aspirin in the treatment regimen of ACS patients reduced cardiovascular events with unstable angina by 46% and proved that antiplatelet medication is considerably advantageous in patients with ACS [5]. Moreover, heparin was the most often prescribed anticoagulant by physicians (57 percent). Furthermore, 25% of all prescriptions are filled with enoxaparin, 7% with rivaroxaban, and 9% of patients received dual anticoagulants. The use of antiplatelet and anticoagulant medication decreases the chance of ACS recurrence and rehospitalization, hence reducing the cost of hospital admissions, which is a significant component of the total cost of managing ACS. Therefore, encouraging clinicians to adhere to the recommended guidelines for the management of ACS will reduce ACS recurrence, hospital admissions, and the total cost of ACS [48].

Nitrates were the most widely recommended coronary artery vasodilators (78 percent). Nitrates dilate and enhance coronary collateral flow in both normal and diseased coronary arteries. Isosorbide dinitrate was the most usually prescribed medicine in the current study (28.5 percent). In addition, in investigations conducted by Muhit et al. (2012) and Bake & Labu (2013), nitroglycerin was the most usually recommended medication [49,50]. Furthermore, amlodipine accounted for 18% of prescriptions, while trimetazidine accounted for 3% of prescriptions. CCBs produce comparable coronary vasodilation and are hence favored in vasospastic angina. Verapamil may be useful in lowering long-term episodes following AMI. The most usually prescribed calcium channel blocker was amlodipine. Swathi et al., 2016 reported similar results [51].

In our study, the most often prescribed antihypertensive medications were beta-blockers (43%), ACE inhibitors (28%), ARBs (16%), CCBs (9%), and diuretics (4 percent). The most prevalent combination was beta-blockers plus ACE inhibitors (33%), followed by beta-blockers plus ARBs (29%), beta-blockers plus CCBs (22%), and beta-blockers plus diuretics (10 percent). Beta-blockers are useful because they reduce myocardial oxygen needs and hence the incidence of ischemia by attenuating the arrhythmogenic potential of the injured myocardium. To prevent mortality in patients with simultaneous ACS, stable heart failure, and diminished systolic function, it is suggested to maintain beta blocker medication with one of three medicines (sustained release metoprolol succinate, carvedilol, or bisoprolol) [5,52]. Unless contraindicated, ACEI is advised indefinitely in all patients with LVEF less than 40%, as well as those with hypertension, diabetes mellitus, and persistent chronic renal disease. ARBs are advised for individuals with heart failure or MI who have LVEF of less than 40% and are ACEI intolerant [5,49,53]. Andhi et al., 2022 and Saju et al., 2020 found comparable results in their research. Valsartan was the most often prescribed ARBs. Metoprolol was the most often prescribed beta-blocker. Furosemide, spironolactone, and torsemide are the most often given diuretics. Amlodipine was the most often prescribed CCB [5,11].

## 5. Conclusions

In conclusion, the present study explored ACS patients’ quality of life and drug prescription patterns at Riyadh region hospitals in Saudi Arabia and documented that all three HRQOL domains were deficient in ACS patients. In addition, statins, antiplatelets, anticoagulants, coronary vasodilators, beta-blockers, diuretics, ARBs, ACE inhibitors, CCBs, and inotropes were the most commonly recommended therapies. Moreover, PCI with CABG reperfusion was used to treat most patients. Additionally, the ACS rate is still rising in Saudi Arabia, especially among younger patients, due to the wide prevalence of uncontrolled risk factors such as smoking, obesity, diabetes, and hypertension, which affect the QOL of the patients. Therefore, the healthcare staff must be educated on the role of QOL in ACS control and prevention and the new ACS management recommendations. To reduce the incidence and effect of ACS, lifestyle changes and evidence-based therapy for CVD risk factors must be prioritized.

## 6. Limitations and Recommendations for Future Studies

Because data were acquired solely in the Riyadh region, the conclusions of this study may not represent the entire population of Saudi Arabia. As a result, more studies with a bigger, multiethnic sample in different regions of Saudi Arabia are required to investigate HRQOL and drug prescription patterns in patients with ACS in Saudi Arabia. In addition, intervention trials to increase HRQOL are required.

## Figures and Tables

**Table 1:** The sociodemographic, symptoms, and diagnostic profiles of ACS patients.

Category	Subcategory	No. of Patients	Percentage	Mean	Standard Deviation	CI (95%)	*p*-Value
**Age**	18–30	31	8.71	25.94	4.00	24.5–27.4	0.002
	31–50	148	41.57	40.25	5.81	39.3–41.2	
	51–70	151	42.42	53.79	4.97	53.0–54.6	
	>70	26	7.30	73.21	3.63	71.8–74.6	
**Gender**	Males	240	67.42	1.33	0.469	0.68–1.98	0.004
	Females	116	32.58				
**Ethnicity**	Rural	86	24.16	1.76	0.429	1.165–2.355	0.001
	Urban	270	75.84				
**Social history**	Smoker	96	26.97	1.73	0.444	1.115–2.345	0.007
	Non-smoker	260	73.03				
**Past history**	Hypertension	169	47.47	1.97	1.131	0.862–3.078	0.006
	Diabetes	134	37.64				
	Neuropsychiatric disorders	43	12.08				
	Renal disorders	25	7.02				
	Respiratory disorders	21	5.90				
**Family history of CVS disorders**	Yes	115	32.30	1.68	0.468	1.031–2.329	0.001
	No	241	67.70				
**Symptoms**	Chest pain	295	82.87				
	SOB	153	42.98				
	Sweating	104	29.21				
	Palpitation and vomit	56	15.73				
	Fever	32	8.99				
	Edema and cough	63	17.70				
**Diagnosis**	NSTEMI	184	51.69	1.67	0.774	0.794–2.546	0.001
	STEMI	105	29.49				
	Angina	67	18.82				
**NYHA classification**	NYHA Ⅰ	96	26.97	1.97	0.713	1.163–2.777	0.002
	NYHA Ⅱ	175	49.16				
	NYHA Ⅲ	85	23.88				
**MRCB scale**	SOB GRADE 0	49	13.76	2.72	1.152	1.71–3.73	0.001
	SOB GRADE 1	119	33.43				
	SOB GRADE 2	103	28.93				
	SOB GRADE 3	52	14.61				
	SOB GRADE 4	33	9.27				

No., number, CI, confidence interval, CVS, cardiovascular system, SOB, shortness of breath, NSTEMI, non-ST-segment elevation myocardial infarction, STEMI, ST-segment elevation myocardial infarction, NYHA, the New York Heart Association scale, and MRCB, Medical Research Council’s Breathlessness scale.

**Table 2:** The MacNew Heart Disease Health-Related Quality of Life Questionnaire.

Category	Subcategory	No. of Patients	Percentage	Mean	Standard Deviation	CI (95%)	*p*-Value
**Emotional functioning**	**Strong**	39	11.0%	2.68	0.993	1.707–3.653	0.001
	**Good**	105	29.5%				
	**Fair**	90	25.3%				
	**Poor**	82	23.0%				
**Physical functioning**	**Mild symptom**	169	47.5%	1.77	0.817	0.845–2.695	0.003
	**Moderate symptom**	100	28.1%				
	**Severe symptom**	87	24.4%				
**Social functioning**	**Dependent**	97	27.2%	1.73	0.446	1.112–2.348	0.002
	**Independent**	259	72.8%				

No, number, and CI, confidence interval.

**Table 3:** Mortality risk in the hospital and at 6 months was calculated using GRACE Scores.

Category	Mortality Risk in Hospital		Mortality Risk at 6 Months	
	No.	Risk Percentage	No.	Risk Percentage
**High risk**	40	11.2%	124	34.8%
**Intermediate risk**	76	21.3%	180	50.6%
**Low risk**	240	67.4%	52	14.6%

**Table 4:** Mortality risk in NSTEMI and STEMI patients was calculated using TIMI Score.

Mortality Risk	No. of Patients		Percentage	
	NSTEMI	STEMI	NSTEMI	STEMI
**High risk**	0	15	0	14.29%
**Intermediate risk**	64	36	35%	34.29%
**Low risk**	120	54	65%	51.43%

No, number, NSTEMI, non-ST-segment elevation myocardial infarction, and STEMI, ST-segment elevation myocardial infarction.

**Table 5:** Diagnosis versus NYHA functional class.

	NYHA Class	Stage 1	Stage 2	Stage 3
**No. of patients**	**NSTEMI**	35	106	43
	**STEMI**	21	50	34
	**Angina**	40	19	8
**Percentage (per total number of patients)**	**NSTEMI**	9.80%	29.80%	12.10%
	**STEMI**	5.90%	14.00%	9.60%
	**Angina**	11.20%	5.30%	2.20%
**Percentage (per total number of patients in stage)**	**NSTEMI**	19.0%	57.6%	23.4%
	**STEMI**	20.0%	47.6%	32.4%
	**Angina**	59.7%	28.4%	11.9%

NYHA, the New York Heart Association scale, No, number, NSTEMI, non-ST-segment elevation myocardial infarction, and STEMI, ST-segment elevation myocardial infarction.

**Table 6:** Diagnosis versus MRCB scale.

		SOB GRADE 0	SOB GRADE 1	SOB GRADE 2	SOB GRADE 3	SOB GRADE 4
**No. of patients**	**NSTEMI**	21	71	60	21	11
	**STEMI**	2	26	39	16	22
	**Angina**	26	22	4	15	0
**Percentage (per total number of patients)**	**NSTEMI**	5.90%	19.94%	16.85%	5.90%	3.09%
	**STEMI**	0.56%	7.30%	10.96%	4.49%	6.18%
	**Angina**	7.30%	6.18%	1.12%	4.21%	0.00%
**Percentage (per total number of patients in grade)**	**NSTEMI**	11.41%	38.59%	32.61%	11.41%	5.98%
	**STEMI**	1.90%	24.76%	37.14%	15.24%	20.95%
	**Angina**	38.81%	32.84%	5.97%	22.39%	0.00%

No, number, SOB, shortness of breath, NSTEMI, non-ST-segment elevation myocardial infarction, STEMI, ST-segment elevation myocardial infarction, and MRCB, Medical Research Council’s Breathlessness scale.

**Table 7:** Commonly prescribed drug classes and Surgical interventions in ACS patients.

Drug Classes	Percentage of Patients	Commonly Prescribed Drugs	Percentage of the Prescribed Drugs
**Statins**	93%	Simvastatin	39%
		Rosuvastatin	33%
		Atorvastatin	18%
		Others	10%
**Antiplatelets**	84%	Aspirin	42%
		Clopidogrel	32%
		Ticagrelor	5%
		Tirofiban	3%
		Others	2%
		Dual Antiplatelet	12%
		Triple Antiplatelet	4%
**Anticoagulants**	79%	Heparin	57%
		Enoxaparin	25%
		Rivaroxaban	7%
		Others	2%
		Dual Anticoagulants	9%
**Coronary Vasodilators**	65%	Nitrates	78%
		Amlodipine	18%
		Trimetazidine	3%
		Others	1%
**Beta-Blockers**	63%	Metoprolol	50%
		Bisoprolol	42%
		Carvedilol	6%
		Others	2%
**Diuretics**	38%	Furosemide	52%
		Torsemide	24%
		Hydrochlorothiazide	12%
		Spironolactone	11%
		Others	1%
**ARBs**	31%	Valsartan	40%
		Telmisartan	26%
		Losartan	19%
		Irbesartan	13%
		Others	2%
**ACE Inhibitor**	22%	Captopril	45%
		Lisinopril	42%
		Perindopril	10%
		Quinapril	2%
		Others	1%
**CCBs**	20%	Amlodipine	76%
		Verapamil	18%
		Nifedipine	4%
		Others	2%
**Inotropes (acute heart failure patients)**	7%	Dobutamine	63%
		Noradrenaline	33%
		Dopamine	4%
**ACS with hypertension**	47.47%	**Solo medication**	15%
		Beta-Blockers	43%
		ACE inhibitors	28%
		ARBs	16%
		CCBs	9%
		Diuretics	4%
		**Combination therapy**	85%
		Beta-Blockers + ACE inhibitors	33%
		Beta-Blockers + ARBs	29%
		Beta-Blockers + CCBs	22%
		Beta-Blockers + Diuretics	10%
		Other combination	6%
**Surgical Intervention**	64%	PCI	49%
		CABG	15%

ARBs, angiotensin receptor blockers, ACE, angiotensin-converting enzymes, CCBs, calcium channel blockers, PCI, percutaneous coronary intervention, and CABG, coronary artery bypass grafting.

## Data Availability

The data presented in this study are available on logic request from the corresponding author.
